# The Effect of PAI-1 4G/5G Polymorphism and Clinical Factors on Coronary Artery Occlusion in Myocardial Infarction

**DOI:** 10.1155/2015/260101

**Published:** 2015-07-26

**Authors:** Tajinder Kumar Parpugga, Vacis Tatarunas, Vilius Skipskis, Nora Kupstyte, Diana Zaliaduonyte-Peksiene, Vaiva Lesauskaite

**Affiliations:** ^1^Institute of Cardiology, Lithuanian University of Health Sciences, Sukileliu 17, LT-50009 Kaunas, Lithuania; ^2^Department of Cardiology, Lithuanian University of Health Sciences, Eiveniu 2, LT-50009 Kaunas, Lithuania

## Abstract

*Objective*. Data on the impact of PAI-1-675 4G/5G genotype for fibrinolysis during myocardial infarction are inconsistent. The aim of our study was to evaluate the association of clinical and genetic (PAI-1-675 4G/5G polymorphism) factors with coronary artery occlusion in patients with myocardial infarction. *Materials and Methods*. PAI-1-675 4G/5G detection was achieved by using Sanger sequencing in a sample of patients hospitalized for stent implantation due to myocardial infarction. We categorized the patients into two groups: patients with coronary artery occlusion and patients without coronary artery occlusion according to angiographic evaluation. *Results*. We identified *n* = 122 (32.4%) 4G/4G, *n* = 186 (49.5%) 4G/5G, and *n* = 68 (18.1%) 5G/5G PAI-1 genotype carriers. Univariate and multivariate analysis showed that only the 4G/5G genotype was associated with coronary artery occlusion (OR: 1.656 and 95% CI: 1.009–2.718, *p* = 0.046). *Conclusions*. Our results showed that carriers of PAI-1 4G/5G genotype with myocardial infarction have increased odds of coronary artery occlusion more than 1.6 times in comparison to the carriers of homozygous genotypes.

## 1. Introduction

Myocardial infarction (MI) in most cases is a result of atherosclerotic plaque rupture. Ruptured plaque triggers blood coagulation cascade and leads to vascular occlusion, reduction of blood flow, and myocardial necrosis. Patients with diabetes mellitus, with excessive weight, with smoking habit, and with arterial hypertension are at increased risk of MI due to thrombosis [1]. Increased risk of coronary artery disease and myocardial infarction is also associated with plasminogen activator inhibitor type I (PAI-1) activity [2]. During injury PAI-1 participates in thrombus stabilization and wound healing processes. PAI-1 is secreted by endothelial cells and is stored and released from the platelets during activation [3]. PAI-1 downregulates the process of fibrinolysis as it stops the conversion of plasminogen to plasmin regulated by both the plasminogen activators: tissue-plasminogen activator and urokinase [4].

PAI-1 is also associated with vascular inflammation [5], atherosclerosis, and metabolic syndrome as its levels in these conditions are elevated. Increased PAI-1 activity was found in atherosclerosis, particularly in people suffering from obesity and diabetes mellitus type II. The levels of PAI-1 are found increased in human CD 34+ cells in diabetic patients with microvascular complications compared to age matched nondiabetic controls [6]. Moreover it was found that increased PAI-1 levels are associated with increased visceral obesity [7] as PAI-1 is produced by ectopic fat depots [8]. In inflammation increase of PAI-1 activity results in altered activity of cytokines (IL-8 and leukotriene B4) and monocyte migration [9]. However, only in rare genetic disorders with lack of PAI-1 activity, prolonged haemorrhagic complications were stated [1].

PAI-1 is a member of serpin (serine protease inhibitors) superfamily of protease inhibitors. There are different PAI-1 polymorphisms of clinical importance described previously by various researchers. The most commonly described ones are the following: PAI-1 (rs1799889) -675 4G/5G insertion/deletion polymorphism at -675 in the promoter region [10], G-A substitution at position -844 (rs2227631) [11, 12], c.43G<A (p.A15T, rs6092), and (p.I17V, rs 6090) [13]. It has been stated that different polymorphisms result in different levels of PAI-1 concentrations. In case of the PAI-1-675 4G/5G, increased levels of PAI-1 in plasma were detected specially for patients carrying 4G/4G genotypes. In another polymorphism named G-A substitution at position -844 (rs2227631) A/A, A/G (A/A carried even more PAI-1 plasma levels than A/G) were involved in higher PAI-1 levels in plasma than the genotype G/G [12]. The polymorphism c.43G<A (p.A15T, rs6092) was also involved in higher PAI-1 plasma levels with A/A, A/G genotypes associated with higher plasma PAI-1 levels than G/G genotypes [13].

PAI-1 is an acute phase enzyme; its activity depends on inflammatory factor stimulation. Until now, the exact effect of the polymorphism PAI-1 (rs1799889) -675 4G/5G (most thoroughly studied and described) on the risk of thrombosis has not been defined clearly. Some studies demonstrated association of PAI-1 4G4G genotype with myocardial infarction [12, 14] while previous studies did not confirm the impact of PAI-1 genotype on thrombolysis during MI [15–17]. The aim of our research was to determine the impact of clinical and genetic (PAI-1-675 4G/5G polymorphism) factors on coronary artery occlusion in the sample of patients with myocardial infarction.

## 2. Materials and Methods

All the procedures used have been reviewed in compliance with ethical standards of the Regional Bioethics Committee of Kaunas, Lithuania, in 2008.05.12 (the permission number is BE-2-30) and with the World Medical Association Declaration of Helsinki on Ethical Principles for Medical Research Involving Human Subjects.

### 2.1. Study Population and Inclusion Criteria

A retrospective analysis was performed in the Molecular Cardiology Laboratory at Cardiology Institute, Lithuanian University of Health Sciences (LUHS), Kaunas. Clinical data and DNA samples of the represented patient population were prepared during the “MI study” from the patients hospitalized in the Department of Cardiology at LUHS from 2008 till 2014 and stored to date. All of these patients were hospitalized for PCI and stent implantation due to acute coronary syndromes (myocardial infarction or unstable angina). Only the patients who followed angiographic evaluation during hospitalization were included into the further analysis.

We categorized the patients into two groups as follows:

1. Patients without coronary artery occlusion, coronary arteries with nonsignificant lesion (defined as less than 50% of visible occlusion).
2. Patients with coronary artery occlusion, patients with nonocclusive coronary artery disease with significant narrowing of coronary vessels due to thrombosis (it takes into account narrowing of >50% (50–99%) vessel diameter due to thrombosis, or patients with total occlusion of coronary arteries (100% reduction in vessel diameter due to thrombosis (no postobstruction antegrade flow is visible on angiography)).

This definition was described according to Sianos et al., 2005 [18].

### 2.2. Genotype Detection and Sequencing

Genotyping procedures were done at the Laboratory of Molecular Cardiology, Institute of Cardiology, Lithuanian University of Health Sciences.

#### 2.2.1. Primary PCR

For genomic DNA sequence detection separate PCR primers were used: upstream primer: 5′-AAGCTTTTACCATGGTAACCCCTGGT-3′ and downstream primer: 5′-TGCAGCCAGCCACGTGATTGTCTAG-3′. PCR conditions were as follows: 95°C for 10 min, followed by 35 cycles at 95°C for 15 sec, 60°C for 45 sec, and 72°C for 45 sec. Final step was performed at 72°C for 10 min.

#### 2.2.2. Purification of Primary PCR Product

Purification of primary PCR product was done by using Invitrogen Pure Link PCR Purification Kit, according to manufacturer's protocol: we added 200 *μ*L of binding buffer B2 with isopropanol to 50 *μ*L of a PCR sample. We centrifuged the sample with a provided column at 10000 ×g for 1 minute. We washed the column with 650 *μ*L of wash buffer (W1). The column was centrifuged again at 10000 ×g for 1 minute. Clean PCR product was incubated at room temperature for 1 minute with 50 *μ*L of elution buffer. Column was centrifuged at 10000 ×g for 2 minutes.

#### 2.2.3. Presequencing PCR

Presequencing reaction mixture (Life Technologies) contained PCR grade water (4 *μ*L), 5x buffer (2 *μ*L), upstream or downstream primer (1 *μ*L), and BigDye Terminator (1 *μ*L). 1 *μ*L of primary PCR product was added to the reaction mixture. PCR conditions were as follows: 96°C for 2 min, followed by 25 cycles at 96°C for 30 sec, 50°C for 15 sec, and 60°C for 4 min.

#### 2.2.4. Purification of Secondary PCR Product

Product was cleaned by using Bigdye Xterminator Purification Kit: to each sample containing 10 *μ*L of product, SAM solution (45 *μ*L) and Bigdye Xterminator solution (10 *μ*L) provided by manufacturer were added. They were then thoroughly mixed for 30 minutes followed by centrifugation for 2 min at 1000 g.

#### 2.2.5. Sequencing

**Figure 1:**
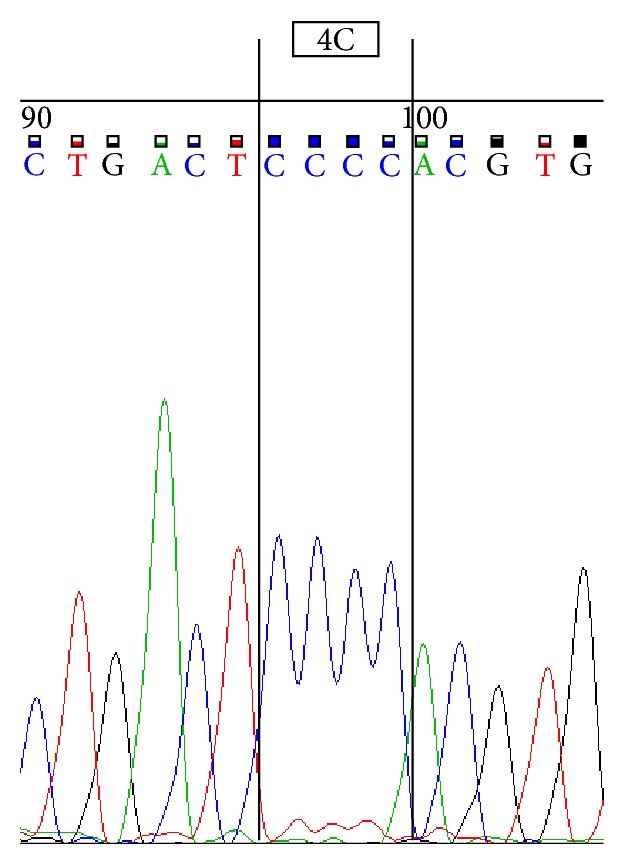
Electropherogram from the sequencing analysis: forward sequences. Homozygous 4G/4G.

**Figure 2:**
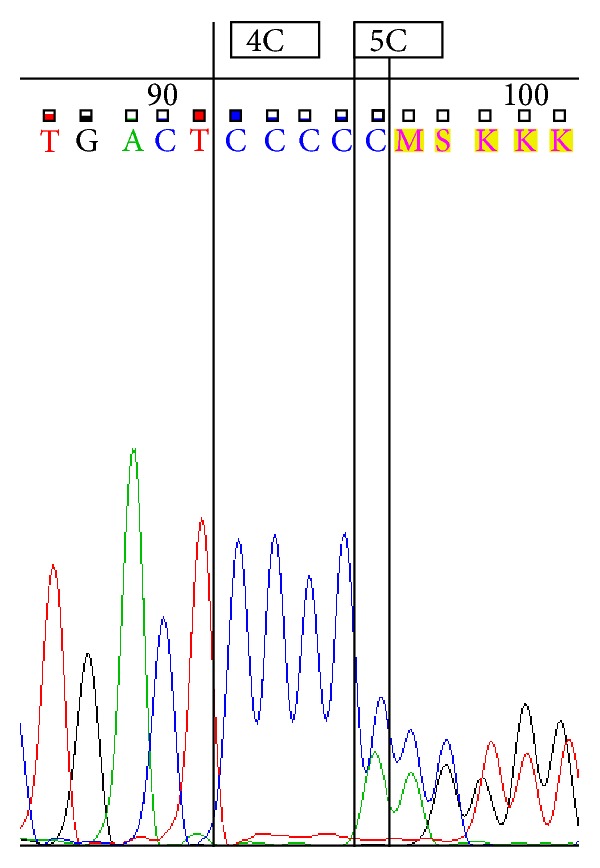
Electropherogram from the sequencing analysis: forward sequences. Heterozygous 4G/5G.

**Figure 3:**
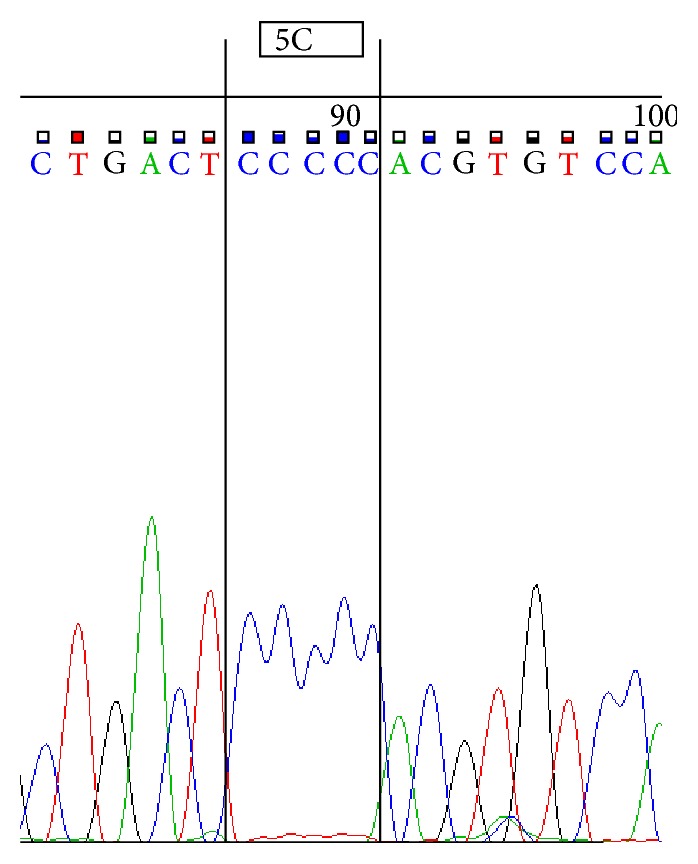
Electropherogram from the sequencing analysis: forward sequences. Homozygous 5G/5G.

**Figure 4:**
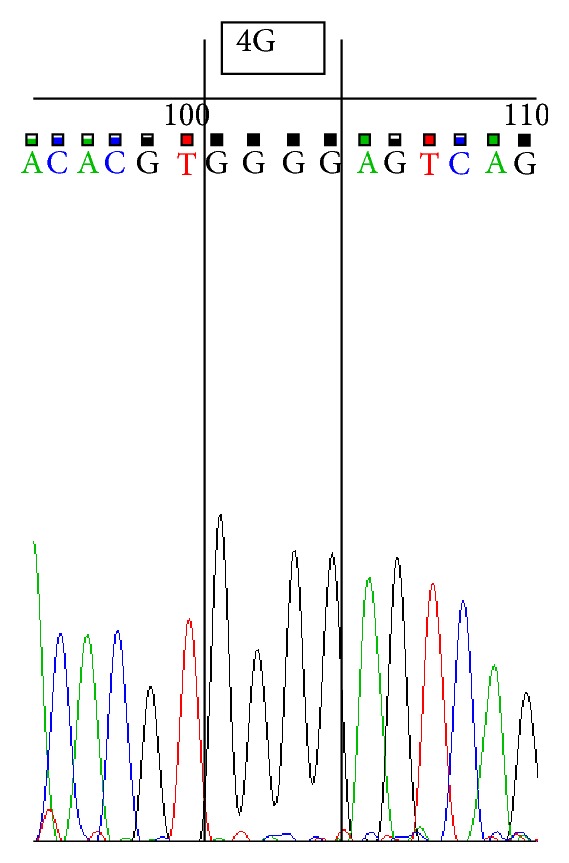
Electropherogram from the sequencing analysis: reverse sequences. Homozygous 4G/4G.

**Figure 5:**
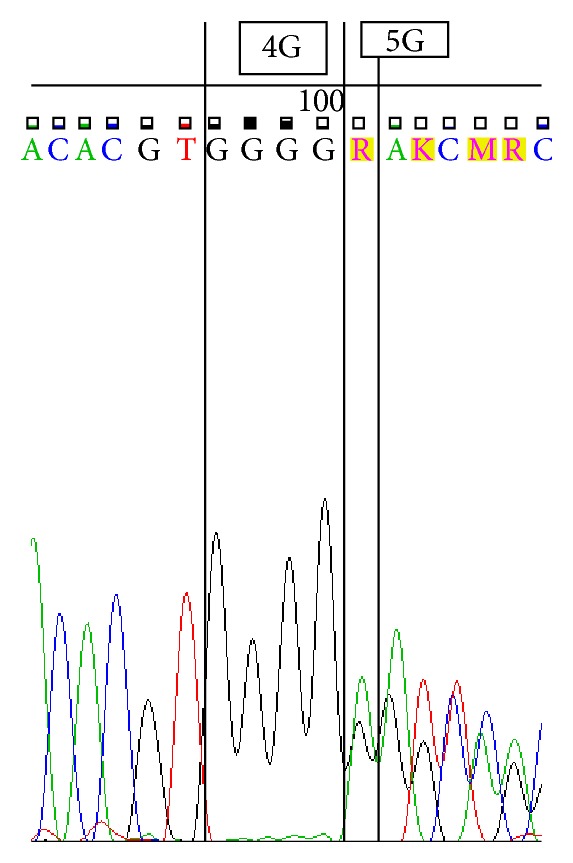
Electropherogram from the sequencing analysis: reverse sequences. Heterozygous 4G/5G.

**Figure 6:**
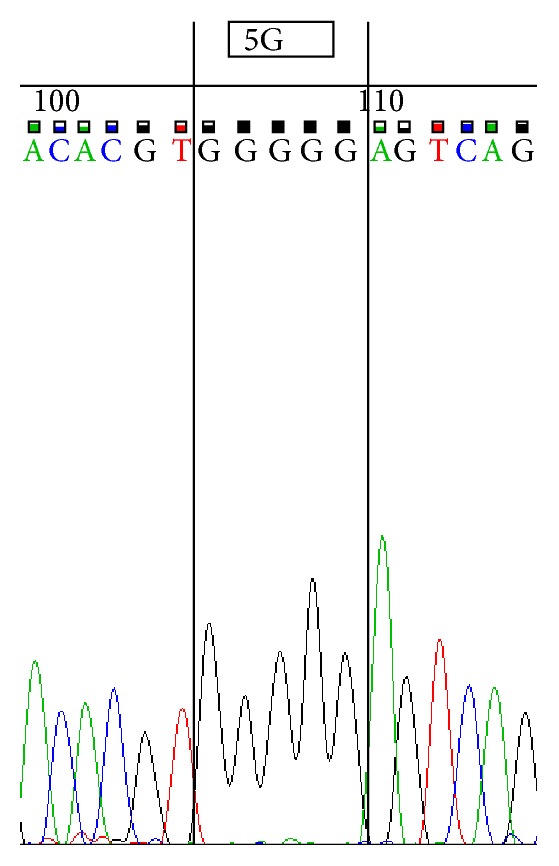
Electropherogram from the sequencing analysis: reverse sequences. Homozygous 5G/5G.

Sequencing was performed by using ABI 3500 Genetic Analyzer according to manufacturer's protocol. Sequence scanner version 1.0 was used to analyze the obtained sequences. We represent sequences determined by using forward primer for 4G/4G (Figure 1), 4G/5G (Figure 2) and 5G/5G (Figure 3) and reverse primer for 4G/4G (Figure 4), 4G/5G (Figure 5), and 5G/5G (Figure 6), respectively.

### 2.3. Statistical Analysis

Frequencies of PAI-1 genotypes are presented in percentages. *χ* ^2^ analysis was used to determine the deviation of allele distribution from the Hardy-Weinberg equilibrium. *χ* ^2^ and Fisher's exact tests were used to analyse categorical data. A binary logistic regression model was used to identify independently associated clinical and genetic factors which significantly determine coronary artery occlusion. First of all we evaluated independent clinical variables by univariate analysis. Recessive (wild type homozygous + heterozygous versus minor allele homozygous), dominant (wild type homozygous versus heterozygous + minor allele homozygous), overdominant (wild type homozygous + minor allele homozygous versus heterozygous), and additive inheritance models were used to calculate the odds ratios (ORs, 95% CI) for PAI-1 4G/5G polymorphism. All variables (clinical and genetic) were taken for multivariable model by backward selection. In the final model we left only those with *p* < 0.05.

## 3. Results

### 3.1. Patient Anthropometric Characteristics

Most of our patients (*n* = 250, 66.5%) were male. Women were older than men, 67.44 ± 10.55 yrs (range 37–86, median 68) versus 60.47 ± 11.00 yrs (range 31–87, median 61), *p* < 0.0001, respectively. Women also had higher body mass index (29.77 ± 5.97) than men (28.23 ± 4.78), *p* = 0.0074.

### 3.2. Impact of Clinical Factors on Coronary Artery Occlusion

Patients with no occlusion of coronary arteries had arterial hypertension more frequently (*χ* ^2^ = 3.894, *p* = 0.048). On the contrary, they had lower prevalence of diabetes (*χ* ^2^ = 3.825, *p* = 0.05) than patients with occlusion of coronary arteries. The factors such as patient gender, body mass index (more than 30 kg/m^2^), or dyslipidaemia (in anamnesis) did not differ significantly between the two groups of patients (Table 1). Men with arterial hypertension (*χ* ^2^ = 4.424, *p* = 0.035) and diabetes (*χ* ^2^ = 4.290, *p* = 0.038) were more prevalent in MI patient group with occlusion of coronary arteries.

### 3.3. Impact of Genetic Factors on Coronary Artery Occlusion

We identified *n* = 122 (32.4%) 4G/4G, *n* = 186 (49.5%) 4G/5G, and *n* = 68 (18.1%) 5G/5G PAI-1 genotype carriers. The PAI-1 alleles of the presented patient group were represented according to Hardy-Weinberg equilibrium (*p* = 0.76) (Table 1).

Univariate analysis showed that only the 4G/5G genotype was associated with coronary artery occlusion (OR: 1.656 and 95% CI: 1.009–2.718, *p* = 0.046). Such factors as arterial hypertension and diabetes showed only a tendency (*p* > 0.05), (Table 2).

Multivariate analysis model added any of important factors on coronary artery occlusion.

## 4. Discussion

In this paper we showed that factors such as patient gender, diabetes, and PAI-1 4G/5G genotype have significant effect on coronary artery occlusion in patients with myocardial infarction. To detect PAI-1 4G/5G genotype, we used Sanger sequencing. It is in contrast to the most of other authors who used allele-specific PCR [11, 19–21], RFLP [12, 14, 22, 23], fragment length detection by using labeled primers [24], or melting curve analysis [25]. Sanger sequencing method for 4G/5G genotype detection is relatively rarely used by other authors [26].

Our sample consisted of patients with MI. We used definitions described by Sianos et al. [18] to classify our patients into groups according to occlusion of coronary arteries. During primary analysis of the data we classified patients into 3 groups as follows: (1) coronary arteries with no occlusion (defined as 0% visible occlusion); (2) nonocclusive coronary artery disease with significant narrowing of vessels due to thrombosis (it takes into account narrowing of >50% (50–99%) vessel diameter due to thrombosis); (3) total occlusion of coronary arteries (100% reduction in vessel diameter due to thrombosis (no postobstruction antegrade flow is visible on angiography)). This analysis revealed that frequency of clinical and genetic variables was similar between 2nd and 3rd groups, so these groups were joined together. More than two-thirds of our studied patients had coronary artery occlusion; it means more than 50% reduction in coronary artery lumen. MI usually has higher prevalence in patients with diabetes and excessive weight, smokers, or patients with elevated blood pressure [1]. While, in the same patients, it differed significantly in terms of coronary artery occlusion, we found that hypertension was more frequently (*χ* ^2^ = 3.894, *p* = 0.048) detected in patients who have no coronary artery occlusion. In addition, our patients without occlusion were less frequently diabetic patients in comparison to the patients with occluded coronary arteries (4% versus 16%, *p* = 0.05, resp.). This was in concordance with one Ukrainian study guided by Efimov et al., which showed that diabetes had an effect on the coronary artery occlusion [27]. Our results did not prove that higher body mass index (more than 30 kg/m^2^) or dyslipidaemia has an impact on coronary artery occlusion.

Large epidemiological studies showed the impact of PAI-1 on MI, as it inhibits fibrinolysis and clot dissolution [1]. During MI, PAI-1 activity is upregulated by renin-angiotensin system and leads to higher prevalence of recurrence of MI in patients, usually in carriers of 4G allele [1]. Other studies also showed that obese women patients with 4G/4G genotypes were at increased risk of thrombotic diseases [28]. Also, the recurrence of MI in normolipidemic postinfarction patients carrying 4G allele was higher [25]. However, 4G/4G genotype prevented greater stenosis formation and might be related to inflammatory activity [14]. A half of our studied patient sample had 4G/5G genotype; one-third of the patients had 4G/4G; only one-fifth of studied sample had genotype 5G/5G. The alleles of PAI-1 matched Hardy-Weinberg equilibrium (*p* = 0.76). Comparison of healthy subjects from other populations revealed that the frequency of 4G/5G genotypes in Lithuanian patient sample was similar to other European populations (Table 3).

Danish study guided by Knudsen et al. [29], French study guided by Collet et al. [30], Belgian study guided by Rapold et al. [31], Italian study guided by Castro et al. [26], and Norwegian study guided by Liguori et al. [32] stated the positive impact of different PAI-1 genotypes on MI due to coronary artery occlusion. However, no significant effect of PAI-1 polymorphism on coronary occlusion has been showed in another study guided by Mehta et al. [33]. By using univariate analysis we also showed that PAI-1 4G/5G genotype was independently associated with coronary artery occlusion in Lithuanian patient sample (OR: 1.656 and 95% CI: 1.009–2.718, *p* = 0.046). Multivariate model of analysis revealed no additional factors that might have an impact on coronary artery occlusion.

Univariate and multivariate analysis models revealed that appropriate application of individualized diagnosis might only be possible if different factors such as clinical and genetic ones are used together.* In conclusion*, our results showed that odds ratios of coronary artery occlusion were increased by 1.6 times in heterozygous PAI-1 4G/5G genotype carriers. It indicates PAI-1 4G/5G genotype as a biomarker for individualized fibrinolysis enhancer use in MI patients.

## Figures and Tables

**Table 1:** The impact of clinical and genetic factors on coronary artery occlusion after MI.

Variable	MI patients with no coronary artery occlusion	MI patients with coronary artery occlusion	Pearson *χ* ^2^, *p*
Gender			
Men, *N* (%)	49 (59.0)	201 (68.6)	
Women, *N* (%)	34 (41.0)	92 (31.4)	
Total, *N* (%)	83 (100.0)	293 (100.0)	2.656, *p* = 0.103
Age in years			
Men, mean ± SD	60.87 ± 9.48	60.37 ± 11.36	
Median (min–max)	63 (36–79)	61 (31–87)	
Women, mean ± SD	65.20 ± 9.79	68.27 ± 10.75	
Median (min–max)	66 (42–79)	70 (37–86)	
Total, mean ± SD	62.65 ± 9.79	62.85 ± 11.74	
Median (min–max)	65 (36–79)	64 (31–87)	
Arterial hypertension			
Men	46 (55.4)	164 (56.0)	4.424, *p* = 0.035
Women	31 (37.4)	83 (28.3)	0.027, *p* = 0.871
Total	77 (92.8)	247 (84.3)	3.894, *p* = 0.048
BMI > 30 kg/m^2^			
Men	15 (18.1)	63 (21.5)	0.010, *p* = 0.921
Women	10 (12.0)	42 (14.3)	2.701, *p* = 0.100
Total	25 (30.1)	105 (35.8)	0.934, *p* = 0.334
Diabetes			
Men	1 (1.2)	24 (8.2)	4.290, *p* = 0.038
Women	4 (4.8)	17 (5.8)	0.806, *p* = 0.369
Total	5 (6.0)	41 (14.0)	3.825, *p* = 0.050
Dyslipidaemia in anamnesis			
Men	40 (48.2)	150 (51.2)	1.379, *p* = 0.240
Women	27 (32.5)	79 (27.0)	0.309, *p* = 0.578
Total	67 (80.7)	229 (78.2)	0.630, *p* = 0.427
PAI-1 genotype distribution according to the patient gender			
Men:			
4G/4G	17 (20.4)	64 (21.8)	2.198, *p* = 0.333
4G/5G	20 (24.1)	103 (35.1)	
5G/5G	12 (14.5)	34 (11.6)	
Women:			
4G/4G	13 (15.7)	28 (9.6)	2.736, *p* = 0.255
4G/5G	13 (15.7)	50 (17.1)	
5G/5G	8 (9.6)	14 (4.8)	
Total			
4G/4G	30 (36.1)	92 (31.4)	4.607, *p* = 0.100
4G/5G	33 (39.8)	153 (52.2)	
5G/5G	20 (24.1)	48 (16.4)	
MAF	0.43	0.42	

MAF: minor allele frequency; BMI: body mass index; SD: standard deviation.

**Table 2:** Univariate and multivariate binary regression analysis for development of coronary artery occlusion.

Variable	Univariate analysis			Multivariate analysis		
	Odds ratio	95 % CI	Significance level, *p*	Odds ratio	95% CI	Significance level, *p*
Age in years	1.002	0.980–1.023	0.884			
Gender (men)	1.516	0.917–2.505	0.104			
Arterial hypertension	2.390	0.983–5.810	0.055			
Diabetes mellitus	2.538	0.969–6.646	0.058			
BMI > 30 kg/m^2^	1.296	0.766–2.193	0.335			
Dyslipidaemia in anamnesis	1.296	0.683–2.459	0.428			
PAI-1	1.237	0.742–2.062	0.415
4G/4G versus 4G/5G + 5G/5G			
PAI-1	0.617	0.342–1.114	0.109
4G/4G + 4G/5G versus 5G/5G			
PAI-1	1.656	1.009–2.718	0.046	1.656	1.009–2.718	0.046
4G/4G + 5G/5G versus 4G/5G						
PAI-1	0.941	0.663–1.334	0.732
5G			

BMI: body mass index.

**Table 3:** Frequencies of PAI-1 4G-675 5G genotypes in different populations of healthy subjects and in Lithuanian patients' sample.

Country	*N*, total	4G/4G		4G/5G		5G/5G		Reference
		*N*	%	*N*	%	*N*	%	
Egypt	48	10	20.8	29	60.4	9	18.8	Ismail et al. [20]
Finland	150	40	27.0	80	53.0	30	20.0	Pastinen et al. [34]
Italy	200	32	16.0	102	51.0	66	33.0	Ardissino et al. [22]
Japan	127	45	35.5	53	41.7	29	22.8	Iwai et al. [35]
Lithuania	376	122	32.4	186	49.5	68	18.1	This study
Mexico	127	17	13.4	38	30.0	72	56.6	Isordia-Salas et al. [23]
Netherlands	302	84	27.8	150	49.7	68	22.5	Doggen et al. [15]
Pakistan	217	52	24.0	89	41.0	76	35.0	Ahmed et al. [19]
Slovenia	145	38	26.2	76	52.4	31	21.4	Stegnar et al. [36]
South Africa	300	65	22.0	132	44.0	103	34.0	Pegoraro et al. [24]
Tunisia	150	36	24.0	65	43.0	49	33.0	Abboud et al. [12]
Turkey	281	73	26.0	112	39.9	96	34.2	Onalan et al. [14]
